# Ewing's Sarcoma of the Upper Extremity: Presenting Symptoms, Diagnostic Delay and Outcome

**DOI:** 10.1080/00207540500050113

**Published:** 2005

**Authors:** Philip M. S. Simpson, Robin Reid, Daniel Porter

**Affiliations:** 1 Department of Orthopaedic Surgery Royal Infirmary of Edinburgh Old Dalkeith Road Edinburgh EH16 4SU Scotland; 2 Department of Pathology Western Infirmary Glasgow Scotland

## Abstract

*Purpose:*To look at the presenting features, Enneking stage, size of primary tumour, method of treatment and patient and doctor delays in upper extremity Ewing's sarcoma to observe the effects on local recurrence, metastasis and survival.

*Patients and methods:*Nineteen patients with upper extremity Ewing's sarcoma were identified using the Scottish Bone Tumour Registry over the past 40 years.

*Results:*With increasing tumour Enneking stage at presentation there was a significantly higher mortality (*P*=0.02). Patients with a higher Enneking stage also had an increased trend towards local recurrence (*P*=0.08). Stage did not influence the occurrence of metastasis. Patients with larger tumours tended to have a higher mortality (50 vs. 27% dead at 5 years). All patients presented clinically with pain and all but two complained of some sort of swelling. There was a trend towards a higher Enneking Stage in patients presenting with a longer duration of symptoms (*P*=0.1). No difference in survival was noted between patients undergoing surgery and chemotherapy and patients undergoing radiotherapy and chemotherapy. Disease-free survival was 100% at both 5 and 10 years for Enneking stage IIA, 56% at 5 and 10 years for stage IIB and 0% at 5 years for stage III.

*Discussion:*This study re-emphasises the potential importance of a diagnostic delay on outcome. Longer symptom duration is associated with a higher Enneking stage at presentation. In turn a higher presenting stage results in a higher mortality. Pain and swelling are prominent clinical findings at first presentation in upper extremity Ewing's.


*Sarcoma*, March/June 2005; 9(1/2): 15–20

Taylor & Francis

Taylor & Francis Group

ORIGINAL ARTICLE

### Ewing's sarcoma of the upper extremity: Presenting symptoms, diagnostic delay and outcome

PHILIP M. S. SIMPSON[1], ROBIN REID[2], & DANIEL PORTER[1]

[1]*Department of Orthopaedic Surgery, Royal Infirmary of Edinburgh, Edinburgh, Scotland, UK*
[2]*Department of Pathology, Western Infirmary, Glasgow, Scotland, UK*

**Abstract**
*Purpose:* To look at the presenting features, Enneking stage, size of primary tumour, method of treatment and patient and doctor delays in upper extremity Ewing's sarcoma to observe the effects on local recurrence, metastasis and survival.
*Patients and methods:* Nineteen patients with upper extremity Ewing's sarcoma were identified using the Scottish Bone Tumour Registry over the past 40 years.
*Results:* With increasing tumour Enneking stage at presentation there was a significantly higher mortality ($P = 0.02$). Patients with a higher Enneking stage also had an increased trend towards local recurrence ($P = 0.08$). Stage did not influence the occurrence of metastasis. Patients with larger tumours tended to have a higher mortality (50 vs. 27% dead at 5 years). All patients presented clinically with pain and all but two complained of some sort of swelling. There was a trend towards a higher Enneking Stage in patients presenting with a longer duration of symptoms ($P = 0.1$). No difference in survival was noted between patients undergoing surgery and chemotherapy and patients undergoing radiotherapy and chemotherapy. Disease-free survival was 100% at both 5 and 10 years for Enneking stage IIA, 56% at 5 and 10 years for stage IIB and 0% at 5 years for stage III.
*Discussion:* This study re-emphasises the potential importance of a diagnostic delay on outcome. Longer symptom duration is associated with a higher Enneking stage at presentation. In turn a higher presenting stage results in a higher mortality. Pain and swelling are prominent clinical findings at first presentation in upper extremity Ewing's.

### Introduction

Ewing's sarcoma is a rare malignant tumour occurring primarily in bone but also not uncommonly in soft tissue. Presenting mainly in the second decade of life it has a predilection for the pelvis, the ribs and the diaphyses of long bones. At the time of presentation, approximately 25% of people have radiological metastases. Prior to chemotherapy the relapse rate was approaching 80%, suggesting systemic spread for many people at the time of diagnosis. The treatment of Ewing's sarcoma currently involves a multidisciplinary approach. The use of four-agent chemotherapy has revolutionised the prognosis in Ewing's sarcoma,[1,2] but some debate still remains as to whether radiotherapy or surgery for localised lesions is superior in terms of survival [3–7].

In the upper extremity, the effects of surgery may have far-reaching consequences in terms of function of the upper limb and hand, particularly in the case of amputation. While there is an increased risk of second malignancy with radiotherapy,[8,9] it has a less disabling impact on function compared to surgery. If there is no survival advantage with surgery for patients developing tumours in the upper extremity then it may be more appropriate to recommend radiotherapy as an adjunct to chemotherapy. In this study the Scottish experience of the presentation and treatment of Ewing's sarcoma in the upper extremity over the last 40 years was analysed to see if any recommendations could be made regarding the most appropriate treatment modalities for these patients.

Analysis was made of the presenting features, Enneking stage [10] size of primary tumour and patient and doctor delays to observe the effects on local recurrence, metastasis and survival.

### Patients and methods

The Scottish Bone Tumour Registry is a clinical, pathological and radiological database on the

Correspondence : Philip Simpson, MRCSEd, Specialist Registrar in Orthopaedic Surgery, Royal Infirmary of Edinburgh, Old Dalkeith Road, Edinburgh EH16 4SU, Scotland, UK. E-mail: drphilsimpson@aol.com

ISSN 1357–714X print/ISSN 1369–1643 online © 2005 Taylor & Francis Group Ltd
DOI: 10.1080/00207540500050113

majority of bone tumours diagnosed in Scotland over the last 40 years. The Registry was used to identify 19 patients who had been treated for Ewing's sarcoma of the upper limb including the shoulder girdle. Data were gathered on patient age, presenting features, radiographic features, time to diagnosis and treatment, type of treatment, complications, and survival. All archival pathology was reviewed by an experienced musculo-skeletal histopathologist to confirm diagnosis.

The chi-square test was used for statistical analysis. The significance level was set at $P<0.05$. Kaplan–Meier statistics were used to calculate survival data.

### Results

Nineteen cases of upper limb Ewing sarcoma were identified from the database, 14 of whom were male and five of whom were female. Their ages ranged from 3 to 57 years, with a mean age of 19 years. Review of the histology confirmed that the recorded diagnosis of Ewing's sarcoma on the database was correct in each case. Details of patient demographics, presenting symptoms, tumour site and follow up can be seen in table 1.

Table I. Patient demographics, presenting features and length of follow-up

| | | |
|---|---|---|
| Number of patients: | 19 | |
| Age range: | 3–57 (mean 19) | |
| Gender: | Male: | 14 |
| | Female | 5 |
| Site: | Shoulder girdle: | 2 |
| | Humerus: | 11 |
| | Forearm: | 4 |
| | Hand: | 2 |
| Presenting Features: | Pain: | 19 |
| | Night pain | 5 |
| | Severe pain | 2 |
| | Swelling: | 17 |
| | Neurological symptoms: | 2 |
| | Loss of Function: | 7 |
| Length of follow-up: | | |
| -All patients: | Range of 6–392 months | |
| | Median 30 months | |
| | Mean 81 months | |
| -Survivors: | Median 149 months | |
| | Mean 165 months | |

One patient lost to follow-up when he returned to the African continent after 6 months disease free

### *Time from symptom onset to first doctor appointment – patient delay*

Patient delay varied from 1 to 180 months with a median of 6 months. When the patient delay was compared with Enneking stage at presentation there was a trend towards a higher stage at presentation with longer symptoms ($P=0.1$).

### *Time from first doctor appointment to definitive diagnosis – doctor delay*

Doctor delay varied from 1 to 128 weeks with a median of 5 weeks.

### Radiographic features

Original radiographic images were available for analysis in 17 of the patients. Eleven patients had tumours which were less than 80 mm in maximum dimension, while six had tumours of greater size than this. Patients with larger tumours tended to have a higher mortality (50 vs. 27% dead at 5 years).

### Treatment

### *Chemotherapy*

Sixteen of the 19 patients underwent neoadjuvant chemotherapy (table 2).

Two others, who were treated in 1965 and 1971, had only radiotherapy for their tumours. Ten patients had four-agent chemotherapy, while six had three-agent chemotherapy. One other patient was given single-agent chemotherapy for palliative purposes.

### *Radiotherapy*

Fifteen of the 19 patients were treated with radiotherapy. Nine had their therapy as part of the primary treatment, while six others underwent radiotherapy for palliative purposes only. All primary radiotherapy treatments were of high dose (45–60 Gy). There were no cases of second malignancies reported in the registry, although one patient was lost to follow-up when he moved back to Africa.

### *Surgery*

Twelve of the 19 patients underwent surgery. Fifteen procedures were carried out in total, and in 10 instances this was performed as part of the primary tumour treatment.

Following inadequate resection margins in three patients, further surgery was performed with two forequarter amputations and a wide local excision of residual clavicle. A forearm amputation was performed palliatively in one patient who had not undergone any primary surgical treatment and one further patient underwent a thoracic laminectomy to palliate spinal metastasis.

### *Chemotherapy and surgery*

Five cases were identified where chemotherapy and surgery were the primary treatment modalities. One was Enneking stage IIA, while the other four

Table II. Year of diagnosis, presenting Enneking grade, treatment and survival

| Year of diagnosis | Age | Gender | Site | Enneking grade | Chemotherapy | Treatment<br>Radiotherapy (dose, Gy) | Surgery | Survival |
|---|---|---|---|---|---|---|---|---|
| 1965 | 7 | F | Proximal humerus | IIA | No | Yes(60) | No | Alive disease-free |
| 1970 | 9 | F | Proximal humerus | IIA | No | Yes(45) | No | Alive disease-free |
| 1972 | 15 | M | Proximal humerus | III | Single agent palliative | Yes(40) | Palliative laminectomy | Died 9 months |
| 1974 | 24 | M | Proximal humerus | IIB | Three-agent | Yes(50) | No | Alive disease-free |
| 1980 | 17 | M | Proximal humerus | III | Three-agent | Yes(60) | No | Died 1 year + 2 months |
| 1980 | 8 | F | Proximal humerus | IIA | Three-agent | Yes(60) | No | Died 1 year + 2 months |
| 1988 | 17 | M | Radius | IIB | Four-agent | Yes(60) | Radial excision | Alive disease-free |
| 1989 | 11 | M | Radius | III | Three-agent | Yes (unknown for palliation) | Forearm amputation | Died 1 year + 4 months |
| 1989 | 3 | M | Proximal humerus | IIB | Three-agent | Yes(50) | No | Alive disease-free |
| 1989 | 12 | M | Radius | IIA | Four-agent | Yes(55) | No | Alive disease-free |
| 1992 | 16 | F | Clavicle | IIB | Four-agent | No | Excision of clavicle | Died 1 year + 2 months |
| 1993 | 28 | M | Phalanx | IIB | Three-agent | No | Triple ray resection (metacarpal) | Alive disease-free |
| 1996 | 17 | M | Phalanx | III | Four-agent | Yes(54) | Ray amputation (metacarpal) | Died 4 years + 3 months |
| 1997 | 31 | M | Proximal humerus | IIB | Four-agent | Yes(55 primarily then 20 + 20 palliative) | Proximal humeral endoprosthesis | Died 1 year + 8 months |
| 1998 | 14 | M | Proximal humerus | IIB | Four-agent | Yes(50) | Humeral resection + fibular graft | Died 2 years + 6 months |
| 1998 | 45 | M | Radius | IIA | Four-agent | Yes(50) | Radial excision | Lost to f/u at 6 months |
| 1999 | 7 | F | Proximal humerus | IIB | Four-agent | Yes(60) | Humeral resection + fibular graft | Alive recurrent disease |
| 2001 | 23 | M | Proximal humerus | IIA | Four-agent | No | Proximal humeral endoprosthesis | Alive disease-free |
| 2002 | 57 | M | Clavicle | IIB | Four-agent | No | Excision of clavicle | Alive disease-free |

were stage IIB. There was one disease-related mortality in the IIB group.

*Chemotherapy and radiotherapy*

Six patients had chemotherapy and radiotherapy as their primary tumour treatment. They tended to have presented with slightly higher Enneking stages of tumour, with two patients staged at grade III, two at IIB and two at IIA. Both patients who had metastases at the time of diagnosis died of their tumours, while one patient in the IIB group died of a chemotherapy-related complication. The patient did not have any evidence of residual disease at autopsy. The two patients with stage IIA disease are still alive.

*Enneking stage at presentation versus metastasis, survival and local recurrence*

The Enneking stage at presentation did not appear to be related to the presence of clinically identifiable metastasis. Higher Enneking stage at presentation was, however, associated with a significantly greater mortality ($P = 0.02$). With regard to local recurrence following treatment, there was a trend towards local recurrence in higher stage tumours ($P = 0.08$).

*Disease-free survival*

Ten-year disease-free survival was 100% for patients with Enneking stage IIA disease and 56% with IIB disease. For stage III disease there were no survivors.

**Discussion**

Despite being the second most common primary bone tumour, Ewing's sarcoma is still rare. Its annual incidence is approximately 0.8 per million population [11]. Even specialist oncological surgeons may see only a handful of cases. Ewing's of the upper extremity is seen even less frequently. This poses particular problems when it comes to identifying patients with a tumour and subsequently deciding the most appropriate management. Previous studies have looked at the presenting symptoms and clinical features of Ewing sarcoma based on data collected at the oncology clinic when a diagnosis was either suspected or confirmed [12,13]. Widhe *et al.* [14] looked at the presenting symptoms and clinical features of Ewing from the first visit to a physician but none of the patients in their series of 48 had a tumour of the upper extremity. In this series, details of the presenting complaints and physical findings found on the first physician visit were also available, so a direct comparison of presenting features was possible between patients with upper extremity Ewing and patients with tumours in other anatomical areas.

In this study tumours occurred almost 3 times as often in males when compared to females, and the mean age was 19 years. These findings concur with previous large studies of Ewing sarcoma. [15,16] All patients presented with pain, and 17 of the 19 (84%) complained of a mass which was palpable on examination. This is greater than the experience of other studies and may be explained by the relative lack of soft tissue coverage in the upper limb. Night pain, which is frequently considered a hallmark of a bone malignancy, was uncommon, occurring in only 26% of cases. The absence of night pain therefore should not be a reassuring feature.

The two patients who complained of neurological symptoms both died and were found to have Enneking stage III tumours at diagnosis. This is, perhaps, not surprising, with features of neural invasion in most tumours also frequently heralding a poor prognosis.

The median diagnostic delay for this series was 35 weeks which is very similar to other published series [14,17,18]. The vast majority of this delay was due to patient factors (30 weeks) and may have occurred due to the intermittent nature of the symptoms described (52% of patients) or the fact that symptom onset was associated with trauma (37% of patients). In one case the doctor-associated delay was particularly prolonged due to an incorrect diagnosis of osteomyelitis being given. This unfortunate pitfall has been described previously. [19] The tendency of patients with Ewing's tumour to have symptoms and signs of sepsis and the necessity for submission of material from any case of suspected bone infection for histology as well as culture cannot be overstated.

The length of symptom duration did not affect the presenting Enneking stage significantly, although it was found that in those who presented with tumours of higher stage there was a significantly higher mortality and a trend towards increased local recurrence.

Increased tumour sizes on imaging was associated with a trend towards increased mortality in accordance with the work of Hayes *et al.*[20] and other published series.

Over time there was considerable heterogeneity in the treatment regimes seen. This was probably as a result of the introduction and improvement of chemotherapy and the fact that little evidence existed regarding the best use of radiotherapy or surgery.

It is interesting to note the difference in incidence between the number of tumours diagnosed in the first 20 years of the registry when compared to the second 20 years (six versus 13 cases). It is not known if this is as a result of a genuine increase in the incidence of these tumours. From 1975 to 1999 the National Cancer Registry has collected information on cases of Ewing's sarcoma in children (0–14 years). [21] During this period 61 cases were recorded in total. Over the same period only 49 cases

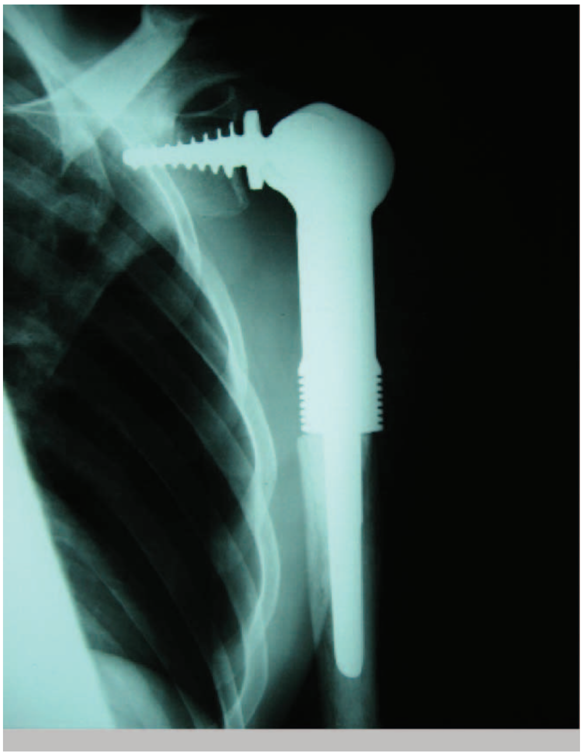

Figure 1. Proximal humeral hinged endoprosthesis used in 23-year-old male.

of Ewing's sarcoma were documented in the Scottish Bone Tumour Registry, suggesting that some of the differences observed in the incidence of Ewing's sarcoma during the 40 year period may be related to incomplete registry. Similar statistics for adults are not available for Ewing's sarcoma in isolation.

The small numbers in this study preclude any meaningful comparison between patients treated with adjuvant radiotherapy or surgery following chemotherapy, although there were no obvious differences between the groups in terms of overall survival. This concurs with the data from the CESS-86 study which found no differences in survival between patients treated with surgery or radiotherapy for localised disease [6,7]. Surgery was found to be superior to radiotherapy in terms of local recurrence, however (11 vs. 20%), with the reverse being the case for metastasis. The most common site of Ewing's in the upper limb is the proximal humerus. Forequarter amputation clearly has far-reaching functional consequences, so a number of limb-sparing surgical strategies have been devised. These involve humeral resection and replacement with either a modular endoprosthesis (Figure 1) or a bulk allograft. If the most proximal and distal segments of the humerus can be preserved then a vascularised autograft is also a possibility. While these have produced good results in terms of elbow, forearm and hand function, the results at the shoulder were generally poor. [22–24] Allografts suffer a high failure rate, while endoprostheses can suffer from painful subacromial impingement secondary to deltoid resection, rotator cuff dysfunction and proximal migration of the prosthesis. Frequently the shoulder ends up stiff.

Radiotherapy offers an alternative treatment to surgery, although it also has its problems. Acutely this may mean skin breakdown or lymphoedema. In the long term, radiotherapy may result in tissue atrophy, fibrosis and muscle contracture, pathological fracture or second neoplasm. In addition radiographs and bone scans can continue to be abnormal for many years following treatment hindering the interpretation of local recurrence. Functional outcome in the upper limb does appear to be better, however [25]. There were no cases of second malignancies in this series. This may due to the relatively short follow-up, with many of the cases having being diagnosed in the last 10 years or the fact that the risk of a second malignancy has been shown to be quite low at 4.7% after 15 years [26].

In conclusion, although the biological behaviour of Ewing's tumours of the upper extremity is similar to Ewing's found in other regions of the body and the presenting symptoms and examination findings are comparable to previous studies, far more upper limb tumours appear to be palpable at the time of presentation. Furthermore, patient delay makes the largest contribution to diagnostic delay and may affect the Enneking stage at presentation. A higher Enneking stage at presentation was found to have a significant effect on survival. The development of an algorithm to alert primary physicians to important worrying features such as swelling may be of value in the upper limb. The question of whether radiotherapy or surgery in addition to chemotherapy is superior in terms of survival remains to be answered. In current practice surgery still seems to be the preferred modality of choice for the control of localised Ewing's tumours.

**Acknowledgements**

The authors would like to acknowledge the help of Jean Campbell, Secretary of the Scottish Bone Tumour Registry, for her help in producing this manuscript.

**References**

1. Nesbit ME, Perez CA, Tefft M, *et al.* Multimodal therapy for the management of primary, non-metastaic Ewing's sarcoma of bone: An Intergroup Study. *Natl Cancer Inst Monogr* 1981;56: 255–62.
2. Bacci G, Picci P, Gherlinzoni F, *et al.* Localised Ewing's sarcoma of bone: Ten years experience at the Instituto Ortopedico Rizzoli in 124 cases treated with multi-modality therapy. *Eur J Cancer Clin Oncol* 1985;21:163.
3. Horowitz ME, Neff JR, Kun LE. Ewing's sarcoma. radiotherapy versus surgery for local control. *Pediatr Clin N Am* 1991;38(2):365–80.

4. O'Connor MI, Pritchard DJ. Ewing's sarcoma. Prognostic factors, disease control and the reemerging role of surgical treatment. *Clin Orthop Rel Res* 1991;262:78–87.
5. Bacci G, Ferrari S, Longhi A, *et al.* Local and systemic control in Ewing's sarcoma of the femur treated with chemotherapy, and locally by radiotherapy and/or surgery. *J Bone Jt Surg Br* 2003;85(1):107–14.
6. Paulussen M, Ahrens S, Dunst J, *et al.* Localised Ewing tumour of bone: Final result of the Cooperative Ewing's Sarcoma Study CESS 86. *J Clin Oncol* 2001;19:1818–29.
7. Dunst J, Hoffman C, Ahrens S, Jurgens H. Surgery versus radiotherapy in Ewing's sarcoma with good prognosis. Analysis of CESS-86 Data. *Strahlenther Onkol* 1996;172(5): 244–8.
8. Tucker MA, D'Angio GJ, Boice JD Jr, *et al.* Bone sarcomas linked to radiotherapy and chemotherapy in children. *New Engl J Med* 1987;317:588.
9. Strong LC, Herson J, Osborne BM, *et al.* Risk of radiation-related subsequent malignant tumours in survivors of Ewing's sarcoma. *J Natl Cancer Inst* 1979;62:1401.
10. Enneking WF, Spanier SS, Goodman M. A system for the surgical staging of musculoskeletal sarcoma. *Clin Orthop* 1980;153:106–20.
11. Vlasak R, Sim FH. Ewing's sarcoma. *Orthop Clin N Am* 1996;27:591–603.
12. Sneppen O, Hansen LM. Presenting symptoms and treatment delay in osteosarcoma and Ewing's sarcoma. *Acta Radiol Oncol* 1984;23:159–62.
13. Wurtz LD, Peabody TD, Simon MA. Delay in the diagnosis and treatment of primary bone sarcoma of the pelvis. *J Bone Jt Surg Am* 1999;81-A:317–25.
14. Widhe B, Widhe T. Initial symptoms and clinical features in osteosarcoma and ewing sarcoma. *J Bone Jt Surg Am* 2000;82-A:667–74.
15. Campanacci M. *Bone and soft tissue tumours*. New York: Springer, 1990;455–63.
16. Arndt CAS, Crist WM. Common musculoskeletal tumours of childhood and adolescence. *New J Med* 1999;341:342–52.
17. Frassica FJ, Frassica DA, Pritchard DJ, Schomberg PJ, Wold LE, Sim FH. Ewing's sarcoma of the pelvis. Clinicopathological features and treatment. *J Bone Jt Surg Am* 1993;75-A:1457–65.
18. Damron TA, Sim FH, O'Connor MI, Pritchard DJ, Smitson WA. Ewing's sarcoma of the proximal femur. *Clin Orthop* 1996;322:232–44.
19. Durbin M, Randall RL, James M, Sudilovsky D, Zoger S. Ewing's sarcoma masquerading as osteomyelitis. *Clin Orthop* 1998;357:176–85.
20. Hayes FA, *et al.* Therapy for localised Ewing's sarcoma of bone. *J Clin Oncol* 1989;7:208.
21. Campbell J, Wallace WHB, Bhatti LA, Stockton DL, Rapson T, Brewster DH. Childhood cancer in Scotland: Trends in incidence, mortality and survival, 1975–1999.
22. Rodi RW, Ozaki T, Hoffman C, Bottner F, Linder N, Winkelmann W. Osteoarticular allograft in surgery for high-grade malignant tumours of bone. *J Bone Jt Surg Br* 2000;82(7):1006–10.
23. Ross AC, Wilson JN, Scales JT. Endoprosthetic replacement of the proximal humerus. *J Bone Jt Surg Br* 1987;69(4): 656–61.
24. Fuhrmann RA, Roth A, Venbrocks RA. Salvage of the upper extremity in cases of tumourous destruction of the proximal humerus. *J Cancer Res Clin Oncol* 2000;126(6):337–44.
25. Kinsella TJ, Loeffler JS, Fraas BA, *et al.* Extremity preservation by combined modality therapy in sarcomas of the hand and foot: an analysis of local control disease-free survival and functional result. *Int J Radiat Oncol Biol Phys* 1983;9: 1115–9.
26. Dunst A, Ahrens S, Paulussen M, *et al.* Second malignancies after treatment for Ewing's sarcoma: A report of the CESS-studies. *Int J Radiat Oncol Biol Phys* 1998;42(2): 379–84.

